# Incidental finding of jejunal diverticula during laparotomy for suspected adhesive small bowel obstruction: A case report

**DOI:** 10.1016/j.ijscr.2021.106268

**Published:** 2021-08-04

**Authors:** Prajjwol Luitel, Bibek Man Shrestha, Shankar Adhikari, Bishnu Prasad Kandel, Paleswan Joshi Lakhey

**Affiliations:** a Maharajgunj Medical Campus, Institute of Medicine, Kathmandu, Nepal; b Department of GI and General Surgery, Tribhuvan University Teaching Hospital, Institute of Medicine, Kathmandu, Nepal

**Keywords:** Adhesiolysis, Complete adhesive small bowel obstruction, Jejunal diverticula, Laparatomy

## Abstract

**Introduction and importance:**

Jejunal diverticula are usually asymptomatic and are discovered incidentally. While rare, their complications may be life-threatening. They should be considered as differential diagnoses in undiagnosed complaints of chronic abdominal pain, malabsorption, anemia, gastrointestinal bleed and intestinal obstruction.

**Case presentation:**

A 66-year lady, known hypertensive and hypothyroidism with history of hysterectomy presented with symptoms suggestive of small bowel obstruction. Intraoperatively adhesions between loops of the small intestine, multiple diverticula with two of them impending perforation were found. Resection of 10 cm of jejunum containing diverticula with end-to-end anastomosis was performed. She had uneventful recovery and on 2 months of follow-up she was doing well.

**Clinical discussion:**

Although diverticula can be found anywhere along the gastrointestinal tract, jejunal diverticula are rare. Most patients are asymptomatic, symptoms if present is non-specific that delay diagnosis causing patients to land up with complications. They are diagnosed incidentally on endoscopy or imaging rather than through clinical suspicion. Asymptomatic cases do not mandate treatment while symptomatic cases can be managed conservatively with surgery being reserved for those with complications.

**Conclusion:**

Small bowel obstruction due to jejunal diverticula is a rare entity, a diagnosis of which can be confirmed only intra-operatively. So it must be borne as a differential in small bowel obstruction. Timely diagnosis and management will prevent life-threatening complications of it.

## Highlights

• Jejunal diverticula arise as a consequence of abnormal motility and increased intraluminal pressure in the jejunum.
• Most patients with jejunal diverticula are asymptomatic. A high index of suspicion is required for their diagnosis.
• Asymptomatic ones don’t require management, while symptomatic ones can be managed conservatively unless complications arise.

## 1 Introduction

Jejunal diverticula are an unusual finding, reported in 0.0006–1.4% of all small bowel contrast studies, 0.3–4.6% of autopsies, and 2.3% of enteroclysis [1]. They are pulsion diverticula thought to be a result of intestinal dyskinesia. Undiagnosed complaints of chronic abdominal pain, malabsorption, anemia, gastrointestinal bleed, intestinal obstruction, distension should borne the differential of jejunal diverticula in mind [2]. Complications include obstruction, perforation, inflammation (less than colonic diverticula due to wider neck at jejunal diverticula), hemorrhage, malignancy [3].

Herein we present an unusual case of multiple jejunal diverticula incidentally found during exploratory laparotomy which was performed for suspected adhesive small bowel obstruction. Thus, timely recognition and management as was done in our case help prevent the complications. This case has been reported in line with SCARE criteria [4].

## 2 Case presentation

A 66-year P_2+1_L_1_ Mongolian female with a prior surgical history of hysterectomy 25 years back but no family history of malignancy presented to the emergency room with a history of lower abdominal pain, loss of appetite, and abdominal distension for 5 days with no passage of stool and flatus for 3 days. There was no complaint of fever, nausea, vomiting, significant weight loss. She used to consume a mixed diet, with no use of alcohol or smoking to date. She was diagnosed with hypertension 10 years back and hypothyroidism 8 years and was under medications.

On examination, she was afebrile with a Blood Pressure of 120/60 mm of Hg, pulse rate of 70 beats per minute, respiratory rate of 16 breaths per minute, and Sp0_2_ of 98% in the room air. On local examination, the abdomen was distended, diffusely tender, with an absence of bowel sounds. Laboratory and biochemical investigations were within normal limits. Plain X-ray finding of dilated proximal small bowel with multiple air-fluid levels and ultrasound revealing extremely gaseous abdomen were suggestive of small bowel obstruction. CT scan was not done due to economic constraint of patient party.

**Fig. 1:**
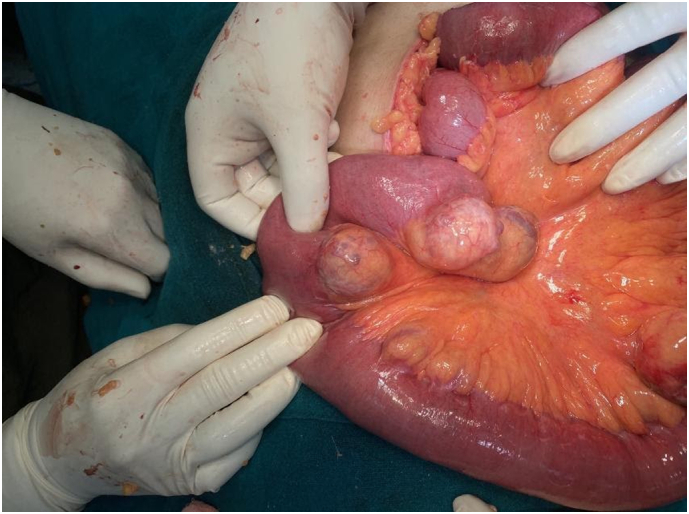
Showing a segment of the jejunum with multiple diverticula on the mesenteric border.

**Fig. 2:**
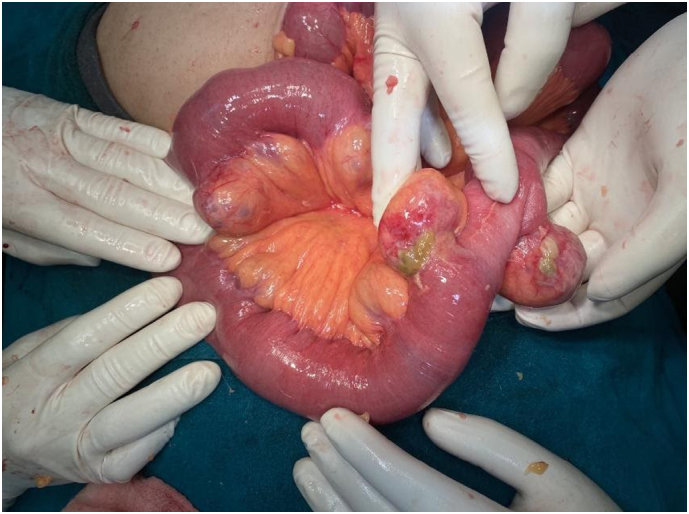
Showing diverticula with impending perforation which were then resected.

**Fig. 3:**
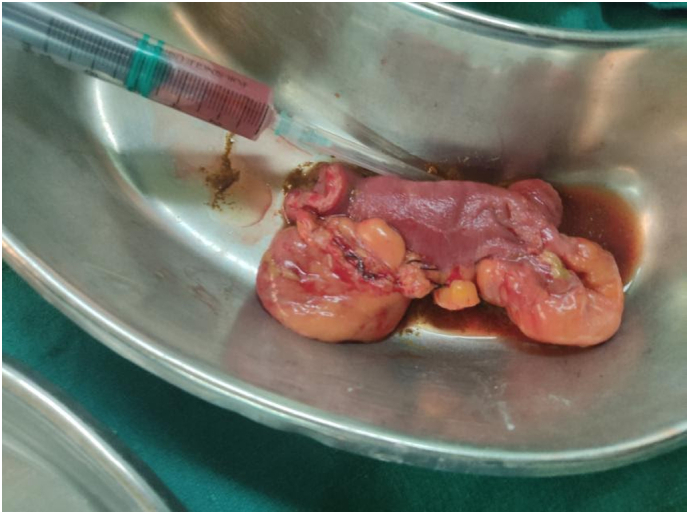
Showing a resected segment of jejunum containing unhealthy diverticula.

With a provisional diagnosis of complete adhesive small bowel obstruction, she was rushed for emergency laparotomy. During surgery, multiple adhesions between loops of the small bowel, with dilation of proximal bowel were found. To our surprise, multiple diverticula in jejunum proximally 10 cm to 45 cm distally from duodenojejunal flexure were found, with two diverticula impending perforation (Fig. 1, Fig. 2, Fig. 3). Extensive search did not show diverticula in small and large bowel. Adhesiolysis was done and around 10 cm of a segment of jejunum containing unhealthy diverticula was resected with end-to-end anastomosis by the team of experienced consultant gastrointestinal surgeons of Tribhuvan University Teaching Hospital. Histological examination of the resected specimen was normal.

Besides surgical site infection which was conservatively managed, the patient had an uneventful recovery. On follow-up after 2 months, she was doing well and was planned for yearly follow-up. Ancillary tests such as colonoscopy were not ordered as the patient was doing completely well during follow-up.

## 3 Discussion

Diverticula are outpouching of hollow or fluid-filled structures. In the gastrointestinal tract, they may arise in the esophagus, stomach, small intestine, gallbladder and colon. In a retrospective review of 208 patients with symptomatic small bowel diverticulosis, diverticula were located in the duodenum in 79%; in the jejunum or ileum in 18%; and in duodenum, jejunum, and ileum in 3% [5]. Jejunal diverticula are protrusion of mucosa through submucosa, muscularis propria on the mesenteric side of the jejunum at the points of entry of the blood vessels, “locus minoris resistentiae” of Edwards [6]. Jejunal diverticulosis is a rare condition with the incidence in intestinal radiography from 0.0006% to 1.4%, at autopsy from 0.3% to 4.6% [1]. Reported incidence in enteroclysis is 2.3% for diverticula of jejunum or ileum or both [7]. The highest incidence occurs in the sixth or seventh decade of life being more commonly reported in men [8]. Soemmering and Baille were the first to describe diverticula in the jejunum in 1794 followed by Astley Cooper in 1809. In 1906 Gordinier and Shil performed the first operation for partial small bowel obstruction, due to inflamed jejunoileal diverticula where resection of the involved jejunal segment was curative [6], [9].

Jejunal diverticula are the consequence of abnormal motility and increased intraluminal pressure due to abnormal structure in either the smooth muscle or myenteric plexus. They are often associated with bowel dysmotility conditions as visceral myopathies, systemic sclerosis and neuropathies [10]. Being deprived of muscularis propria, they are atonic dilatations arising in the mesenteric border in contrast to Meckel's diverticulum in the antimesenteric border [11]. The number of jejunoileal diverticula decreases as we move distally from the ligament of Treitz to ileocaecal valve with nearly 80% of them occurring in the jejunum, 15% in the ileum, and 5% in both [9]. They are multiple, arising in a cluster mostly localized to proximal jejunum [12].

Most patients with jejunal diverticula are asymptomatic. Only 10%–40% of patients with jejunal diverticulosis are reportedly symptomatic. Cooke et al., however, reported symptoms in 90% of their 33 patients [1]. Symptomatic ones present with early satiety, bloating, anorexia, chronic mild abdominal discomfort, diarrhea/steatorrhea due to bacterial overgrowth, colicky pains in epigastrium, peri-umbilical region, loud rumbling noises in the abdomen following meals [2]. The triad of obscure pain, dilated loops of bowel on barium x-ray and anemia is suggestive of jejunoileal diverticulosis [12]. Among them, our patient presented with the first two. Up to 10% of patients with jejunoileal diverticula develop complications [13]. Complications include acute diverticulitis, general peritonitis, localized abscess, adhesions, obstruction, concretion formation, gastrointestinal bleeding, traumatic rupture, volvulus, macrocytic anemia with or without steatorrhea (due to intestinal hypomotility resulting in stagnant loop syndrome). Cooke et al. documented disturbance of vitamin B12 metabolism or absorption in 16/33 patients, and neuropathy in 12/33 patients, both of which were absent in our patient. The most common reported complication is intestinal pseudo-obstruction likely due to jejunoileal dyskinesia than diverticulum itself [14]. Rare complications include fistula formation, neoplasm which includes lipoma, fibroma, carcinoid, sarcoma, and carcinoma [15]. Foreign body impaction and infestation with intestinal parasites have also been noted [3].

Small bowel diverticulosis is often diagnosed incidentally in patients undergoing upper endoscopy or imaging for upper gastrointestinal symptoms [16]. Plain film radiographs of the abdomen will suggest the diagnosis from the presence of air-fluid levels in multiple diverticula throughout the small intestine. Contrast studies (such as upper gastrointestinal series with small bowel follow-through or CT/MR enterography), including enteroclysis of the jejunum and ileum, will reveal a large outpouching with retained contrast medium after the main lumen has become empty [17]. The lumen of the intestine will be dilated in the diverticula, and the mucosal folds will be thickened and prominent. Complications may also be visualized in CT/MRI [12].

Management of jejunal diverticula relies in it's presentation. Asymptomatic ones do not call for any treatment. In symptomatic ones with complaints of chronic vague abdominal symptoms, diarrhea, malabsorption, conservative measures as low-residue high-protein diet, vitamin supplementation, broad-spectrum antibiotics, tetracycline or erythromycin, ampicillin, and metronidazole, antidiarrheal agents, proton-pump inhibitors, antispasmodics, and analgesics are used unless large diverticula with dilated hypertrophied loops of small bowel are discovered [12]. Restricted diet and antibiotics suffice for management of an acute uncomplicated diverticulitis. Nonetheless, if the patient lands with complications, the resection (laparoscopic or open) of the affected segment of the small bowel with primary end-to-end anastomosis is the procedure of choice, the exception being panjejunoileal diverticulosis where conservative treatment may be preferable [16]. However only resection of diverticula can also be done. Overlooking of diagnosed diverticula can advance to recurrent massive gastrointestinal bleeding and death [18]. They are hidden between the folds of the mesentery, collapsed so may be easily missed unless specifically explored for [9]. Complications of diverticular bleeding demands resuscitation and if it does not halt spontaneously, identification and treating the bleeding site. Bowel obstruction due to enterolith impaction in diverticula is treated with enterotomy, stone extraction, manual crushing, or milking of it [19]. Pseudo-obstructive symptoms may not be relieved if the remaining bowel without diverticula is with an underlying neuromuscular disorder. In diagnostic uncertainty, surgery may be performed to outrule the mechanical cause of obstruction and to get tissue for histopathological diagnosis [6], [20].

Features of small bowel obstruction in our patient were either due to adhesions due to the previous hysterectomy or due to multiple jejunal diverticula remains still unknown. The diverticula in our patient were not associated with diverticula elsewhere in the gastrointestinal tract which is said to be common in other studies. Our patient was totally fine during her followup so invasive imaging were not advised. Had it not been detected on laparotomy it could have perforated and had the patient presented with complications, the management would have been more arduous, therefore high suspicion and early diagnosis are of utmost importance in this entity. Recurrence of symptoms is more common in the group presenting with symptoms (53% (21/39)) than in the one in which it is incidentally found (17% (6/34)) [9]. On follow-up of our patient for 6 months, she did not have any bowel symptoms suggestive of recurrence of jejunal diverticula.

## 4 Conclusion

Jejunal diverticula, although an uncommon clinical entity, may cause chronic nonspecific abdominal symptoms and serious acute complications with advancing age, and must be thought of as a differential diagnosis. Asymptomatic ones do not need treatment while symptomatic ones need conservative management unless complications develop which demands surgery.

## Patient perspective

Patient and patient party were satisfied with the care, treatment they received during hospital stay and were thankful to all the health care workers.

## Informed consent

Written informed consent was taken from patient for the publication of case report and accompanying images. A copy of written consent is available for review by the Editor-in-Chief of this journal on request.

A written consent was obtained from patient after she was explained about the purpose of study.

## Credit authorship contribution statement

Conception and design of study: Bishnu Prasad Kandel, Paleswan Joshi Lakhey, Prajjwol Luitel, Shankar Adhikari

Acquisition of data: Prajjwol Luitel, Shankar Adhikari

Analysis and/or interpretation of data: Shankar Adhikari

Drafting the manuscript: Prajjwol Luitel, Shankar Adhikari, Bibek Man Shrestha

Revising the manuscript critically for important intellectual content: Paleswan Joshi Lakhey, Bibek Man Shrestha

Approval of the version of the manuscript to be published: Prajjwol Luitel, Shankar Adhikari, Bishnu Prasad Kandel, Paleswan Joshi Lakhey, Bibek Man Shrestha.

## Declaration of competing interest

The authors declare they have no conflicts of interest.
